# Supplementary material for: CEACAM1 regulates the IL-6 mediated fever response to LPS through the RP105 receptor in murine monocytes
Source: BMC Immunol. 2019 Jan 23;20:7. doi: 10.1186/s12865-019-0287-y (PMC6345024; doi:10.1186/s12865-019-0287-y)
Supplement: Supplementary file 1 — Figure S1. IL-6 mRNA expression in peritoneal cavity tissues after LPS challenge. Figure S2. Intracellular IL-6 and TNFα staining of hepatocytes and Kupffer cells in response to LPS in Ceacam1−/− mice. Figure S3. IL-6 and TNFα levels of liver cells after LPS treatment in vitro and IL-6 receptor downstream signaling activation after i.p. LPS in vivo. Figure S4. CEACAM1 expression on bone marrow CD115+ (M-CSF+) cells and CD115+ cell pattern change of bone marrow cells after treated with LPS + BFA. Figure S5. Macrophages are not involved in the early IL-6 response (< 2 h) to LPS. Figure S6. RAW264.7 cells start to produce IL-6 after treatment with LPS + BFA for 11 h, while silencing of CEACAM1 does not affect IL-6 production in murine macrophage RAW264.7 cells at the 2 h and 24 h time points. Figure S7. Murine macrophage cell line J774A.1 does not produce IL-6 within 5 h after LPS treatment, while silencing of CEACAM1 does not interfere with IL-6 production after LPS treatment at 2 h and 24 h points. Figure S8. PTP inhibitor III, a SHP1 inhibitor, increases levels of phospho-VAV1 in bone marrow monocytes of WT mice. Figure S9. CEACAM1, TLR4 and RP105 expression on human peripheral blood monocytes. (PDF 1828 kb) [file 12865_2019_287_MOESM1_ESM.pdf]

Regulation of the IL-6 mediated fever response to LPS in murine monocytes is regulated by CEACAM1 and the RP105 receptor

Zhifang Zhang*, Deirdre La Placa, Tung Nguyen, Maciej Kujawski, Keith Le, Lin Li, and John E. Shively*

Department of Molecular Imaging and Therapy,
Beckman Research Institute of City of Hope
1500E Duarte Road, Duarte, CA91010, USA
*Correspondence: zzhang@coh.org ; jshively@coh.org

Supplementary Figures and Figure Legends

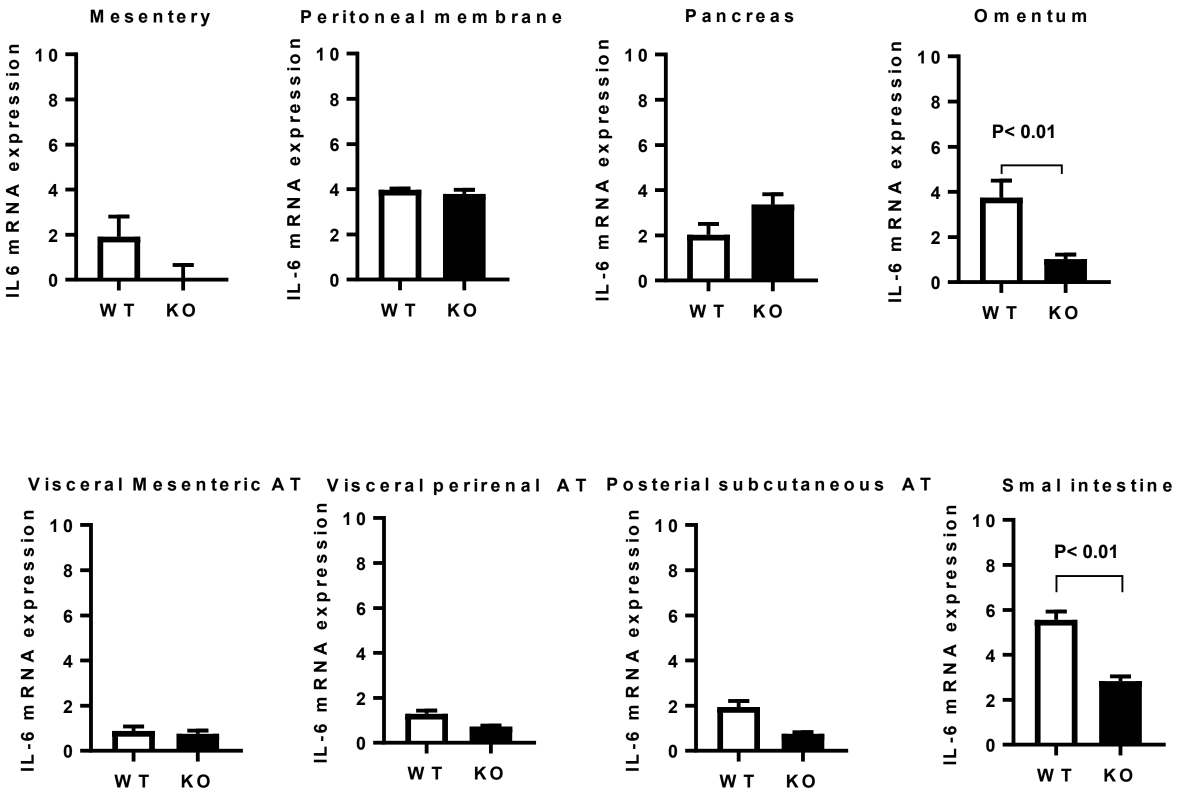

Figure S1. *IL-6* mRNA expression in peritoneal cavity tissues after LPS challenge. *IL-6* mRNA expression levels of indicated tissues by RT-PCR after i.p. injection of LPS (10 mg/kg) for 2 hours (n=4). AT= adipose tissue.

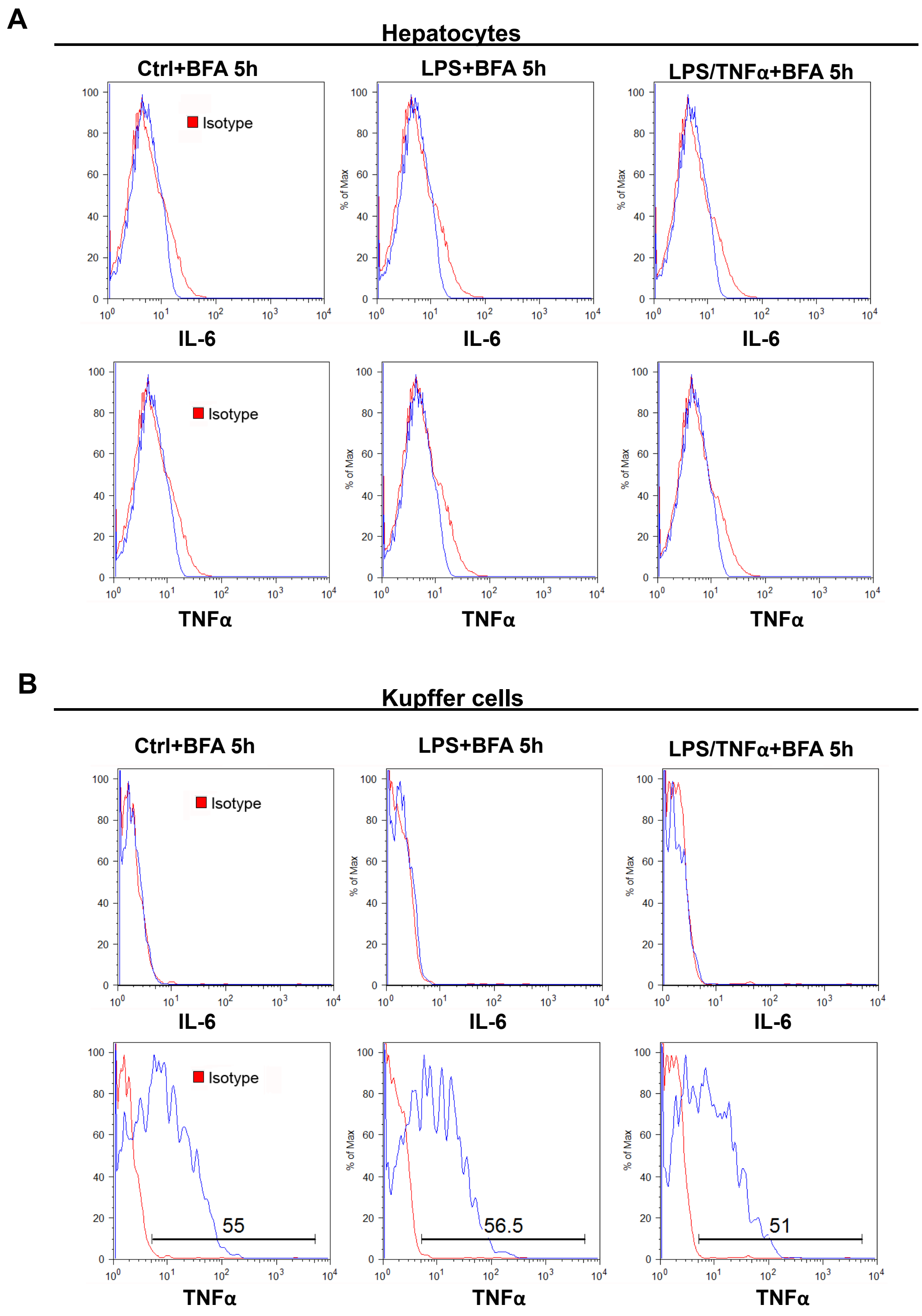

Figure S2. Intracellular IL-6 and TNFα staining of hepatocytes and Kupffer cells in response to LPS in *Ceacam1*$^{-/-}$ mice.

(A) Intracellular IL-6 and TNFα staining of hepatocytes after treatment with Brefeldin A plus LPS (500ng/mL) or LPS+TNFα (20ng/mL) for 5 hours (n=3).

(B) Intracellular IL-6 and TNFα staining of Kupffer cells after treatment with BFA plus LPS (500ng/mL) or LPS+TNFα (20ng/mL) for 5 hours (n=3).

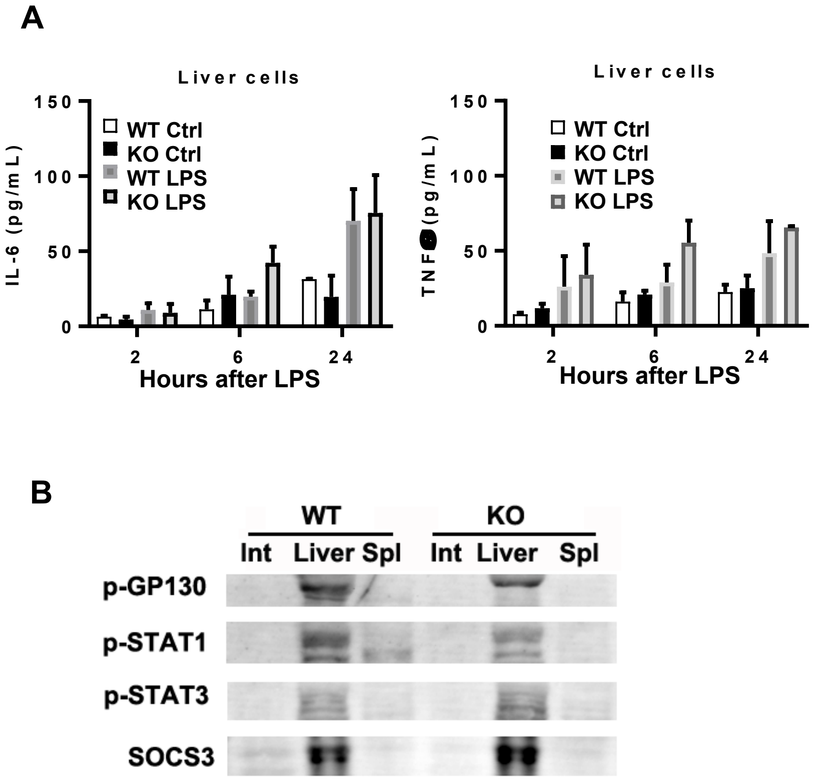

Figure S3. IL-6 and TNFα levels of liver cells after LPS treatment in vitro and IL-6 receptor downstream signaling activation after i.p. LPS in vivo

(A) IL-6 and TNFα release from liver cells after treatment with LPS (500ng/mL) over time in vitro (n=3).

(B) immunoblot of pGP130, pSTAT3, pSTAT1 and pSOCS1 in small intestine (Int), liver and spleen (Spl) after i.p. 10mg/mL LPS treatment for 2 hours in vivo (n=3).

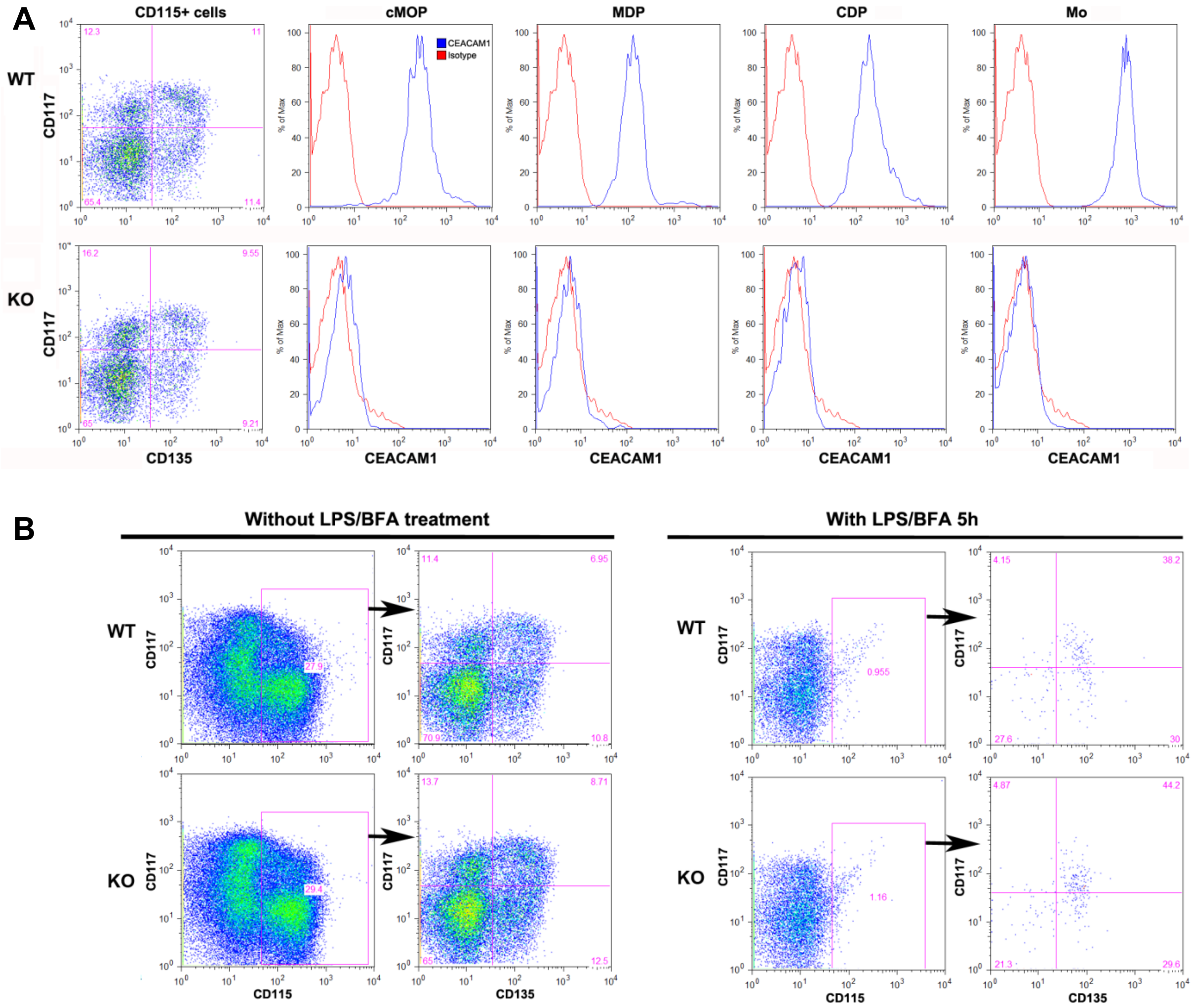

Figure S4. CEACAM1 expression on bone marrow CD115⁺ (M-CSF⁺) cells and CD115⁺ cell pattern change of bone marrow cells after treated with LPS + BFA.

(A) Surface CEACAM1 staining of monocytes (Mo), common monocyte progenitors (cMoP), monocyte-macrophage DC progenitors (MDP) and conmmon DC precursors (CDP) of CD115+ bone marrow cells in WT and *Ceacam1*$^{-/-}$ (KO) mice (n=4).

(B) Flow analysis of CD115, CD117, and CD135 of bone marrow cells treated with LPS +BFA for 5 hours in vitro (n=3).

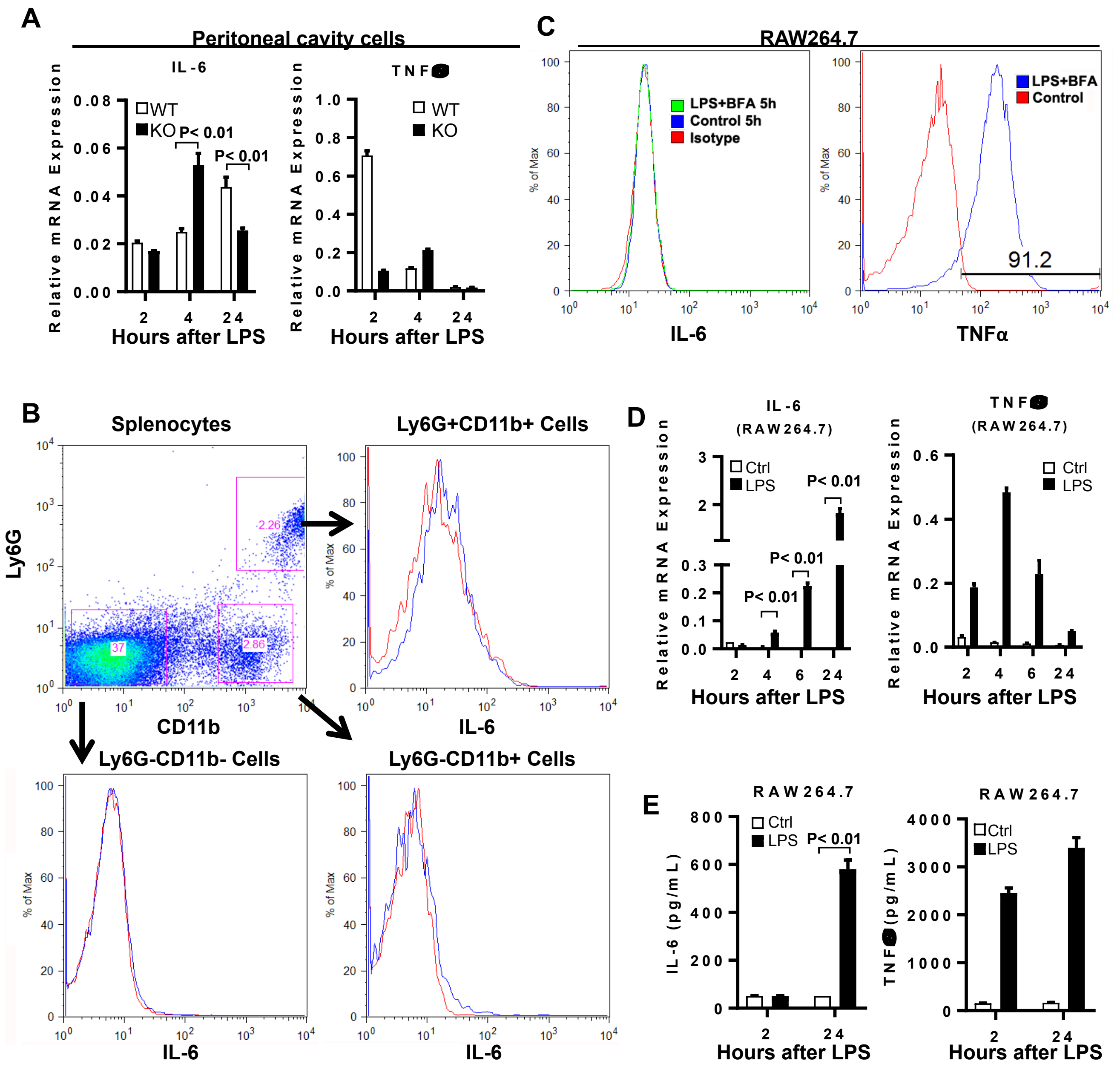

Figure S5. Macrophages are not involved in the early IL-6 response (<2 hours) to LPS.
(A) *IL-6* mRNA and *TNFα* mRNA expression levels in peritoneal cavity macrophages treated with 500ng/mL LPS in vitro (n=3).
(B) Intracellular staining of IL-6 in the splenocytes of *Ceacam1*$^{-/-}$ mice treated with Brefeldin A (BFA) plus LPS for 5 hours in vitro (n=3).
(C) Intracellular staining of IL-6 and TNFα in RAW264.7 cells treated with BFA plus LPS for 5 hours in vitro (n=3).
(D) *IL-6* mRNA and *TNFα* mRNA expression levels in RAW264.7 cells treated with 500ng/mL LPS over time (n=3).
(E) IL-6 protein level in supernatant of RAW264.7 cells treated with 500ng/mL LPS in vitro (n=3).

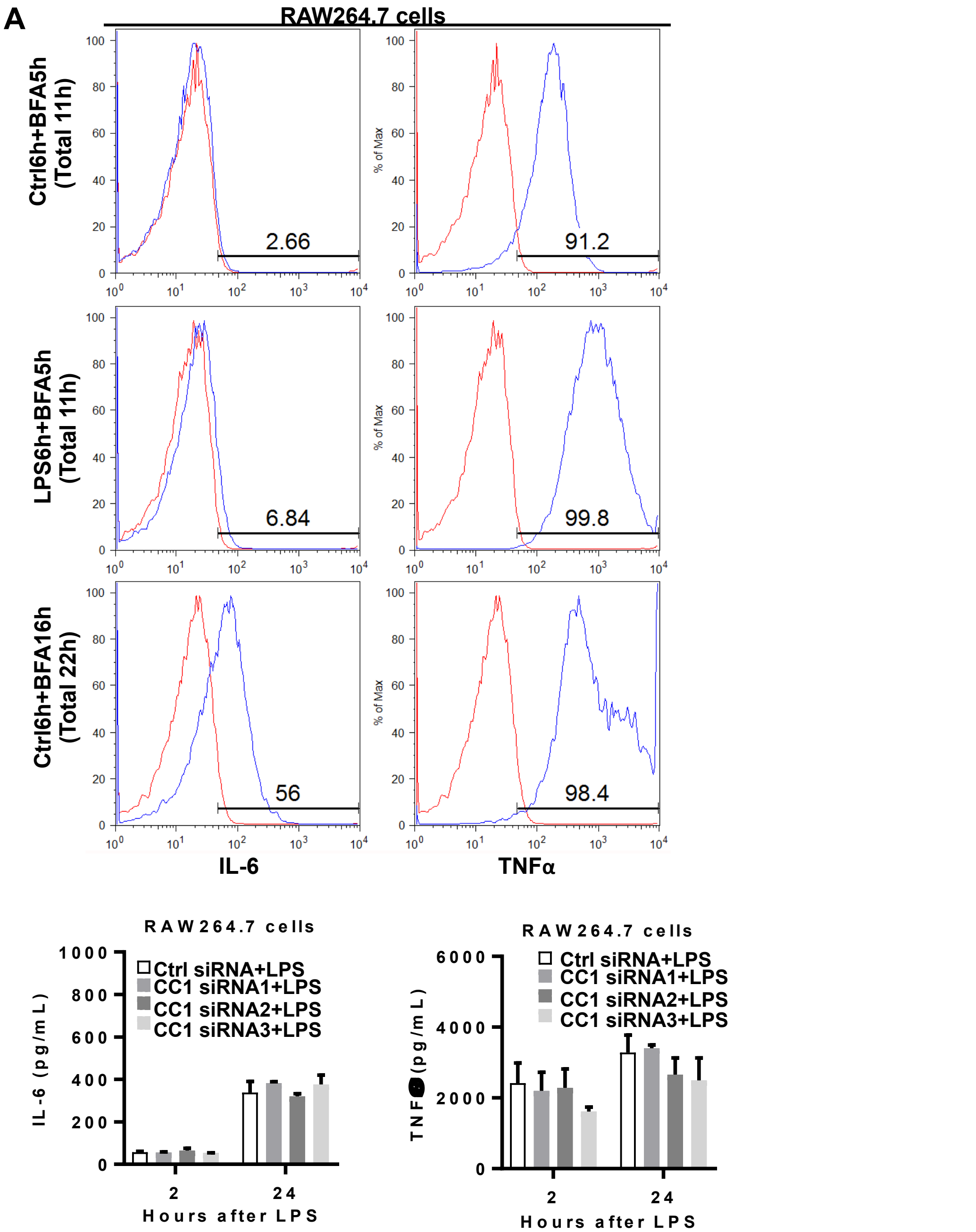

Figure S6. RAW264.7 cells start to produce IL-6 after treatment with LPS+BFA for 11 hour, while silencing of CEACAM1 does not affect IL-6 production in murine macrophage RAW264.7 cells at the 2 hour and 24 hour time points.
(A) Intracellular IL-6 and TNFα staining of RAW264.7 cells after treated with LPS over time shown in the figure (n=3).
(B) Silencing of CEACAM1 by RNAi does not affect IL-6 and TNFα production in murine macrophage RAW264.7 cells at the 2 hr and 24 hr time points (n=3).

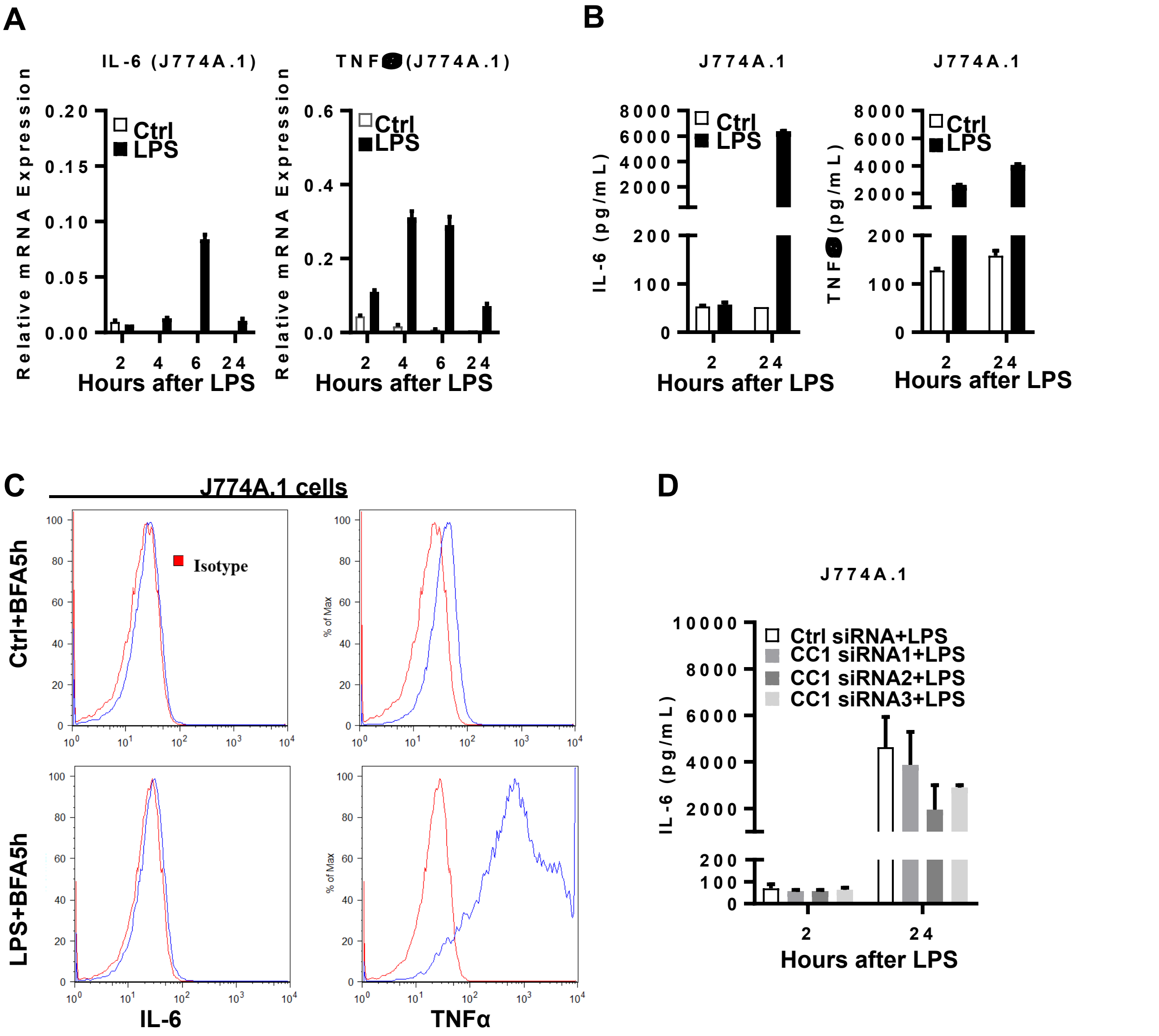

Figure S7. Murine macrophage cell line J774A.1 does not produce IL-6 within 5 hours after LPS treatment, while silencing of CEACAM1 does not interfere with IL-6 production after LPS treatment at 2 hour and 24 hour points.

(A) IL-6 and TNFα mRNA expression after treatment with LPS (n=3).

(B) IL-6 and TNFα level after treatment with LPS (n=3).

(C) Intracellular staining of IL-6 and TNFα of J774A.1 cells treated with LPS plus BFA for 5 hours (n=3)

(D) IL-6 levels of J774A.1 cells treated with LPS over time in which CEACAM1 was silenced by RNAi (n=3).

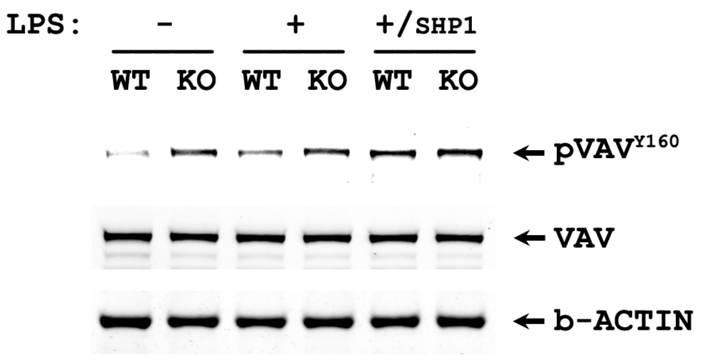

Figure S8. PTP inhibitor III, a SHP1 inhibitor, increases levels of phospho-VAV1 in bone marrow monocytes of WT mice.

Immunoblot analysis of pVAV1 and VAV1 in WT and CEACAM1 KO bone marrow monocytes pretreated with 193uM PTP inhibitor III for 20 minutes following 500ng/mL LPS treatment for 15 minutes.

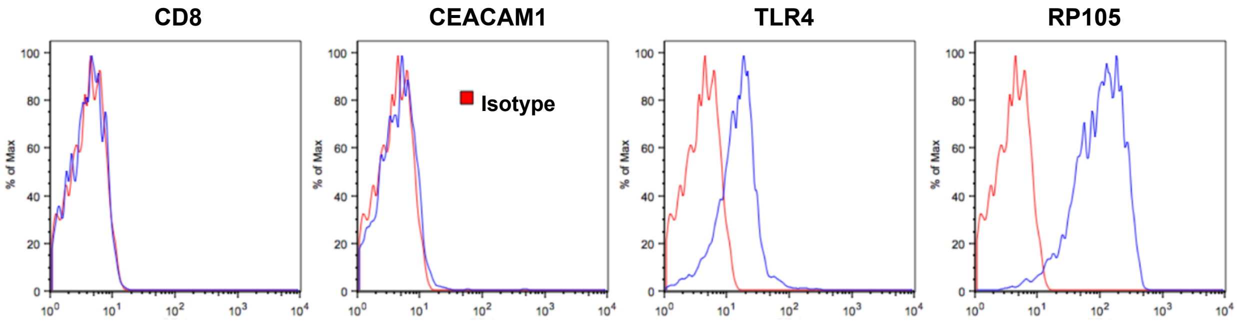

Figure S9. CEACAM1, TLR4 and RP105 expression on human peripheral blood monocytes.
Surface CD8 (negative control), CEACAM1, TLR4 and RP105 staining of CD14 positive monocytes of peripheral blood.
